# Relationships between Alternative Nurse Staffing Level Measurements and Nurses' Perceptions of Nurse Staffing Level Adequacy, Fatigue, and Care Quality

**DOI:** 10.1155/2023/6060536

**Published:** 2023-08-17

**Authors:** Kyung Jin Hong, Hyesook Chung, Young Mi Jo

**Affiliations:** ^1^College of Nursing, Kangwon National University, Chuncheon 24341, Republic of Korea; ^2^Nursing Department, Kangwon National University Hospital, Chuncheon 24289, Republic of Korea

## Abstract

**Aims:**

This study examined the influence of nurse staffing level, measured using various methods, on nurses' perceived adequacy of nurse staffing level, fatigue, and nursing care quality.

**Background:**

Although previous studies have recommended various methods of measuring nurse staffing level, there is a lack of research that compares different measurement methods or considers nurses' perceptions of staffing level on a daily basis.

**Methods:**

We conducted a cross-sectional study using work sampling and the questionnaire method in a general hospital in South Korea from July 18 to August 14, 2022. Results based on responses from 90 nurses and scores of 5,536 inpatients derived from the Korean Patient Classification System were included in the analysis.

**Results:**

The average nurse-to-patient ratio a day was 1 : 3.20, and the registered nursing hours per patient day was 2.35. Perceived insufficient nurse staffing and fatigue were higher on weekdays than on weekends (*p* < 0.001). All variables measuring the nurse staffing level affected the nurses' perceived inadequacy of nurse staffing level, fatigue, and nursing care quality, compared to other variables related to nurse staffing level, such as work intensity and demanding nursing hours per nurse (*R*^2^: 0.19–0.31), the nurse-to-patient ratio had the lowest explanatory power in explaining the nurses' perceptions (*R*^2^: 0.14–0.18).

**Conclusions:**

Nurse staffing level measurement should consider the acuity of inpatients and nursing care time. Further research is needed to utilize nurses' perceptions of the appropriate nurse staffing level. *Implications for Nursing Management*. Efforts are required to maintain an appropriate nurse staffing level through continuous monitoring of nurses' perceptions and acuity of inpatients to preserve nurses' alertness during work and improve nursing care quality.

## 1. Introduction

Previous studies have examined the relationship between adequate nurse staffing and patient outcomes such as inpatient mortality, fall incidences, hospitalization days, and incidences of pneumonia in patients after surgery [1–3]. Although adequate nurse staffing level is an important factor for patient safety, the number of practicing nurses per 1,000 persons in the South Korean population was found to be lower than the average rates reported in other member countries of the Organisation for Economic Co-operation and Development (OECD) [4]. Moreover, the declined inpatients' length of stay and increased average acuity of patients have made the care burden heavier and increased the demand for nursing staff [5]. The workload of registered nurses and nurse staffing level differs between weekdays and weekends in South Korea [6].

As nurse staffing is a factor that can strongly influence the quality of care [7], scholars have developed methods of measuring this variable and attempted to identify the characteristics of optimal nurse staffing [8, 9]. As nurse staffing measurement systems, previous studies have mainly used nurse-to-patient ratios, the acuity/dependency method, nursing hours per patient day (NHPPD) or registered nursing hours per patient day (RN HPPD), and nurse-perceived staffing adequacy [10–12]. The nurse-to-patient ratio is a relatively simple and quick calculation method that tends to allocate nursing resources without regard for patient need or complexity [13]. A policy in South Korea limits the nurse-to-patient ratio to 2.5 or less, and 12 outpatients are counted as one inpatient [14].

Moreover, the acuity/dependency method relies on patients being classified based on acuity and dependency, according to which nursing requirements are then determined [15]. The Safer Nursing Care Tool is the most widely used method; acuity refers to patients' increased risk of clinical deterioration and complexity, whereas patient dependency means the level of need to support their physical activities, such as eating, drinking, and personal hygiene [16]. Patient dependency values can be used to estimate the work intensity of nurses; an example is the Oulu Patient Classification (OPC), developed in Finland [17, 18]. NHPPD or RN HPPD refers to the total number of productive hours worked by nursing staff or registered nurses per patient day on a designated inpatient unit during a specific calendar month. It has been widely used because of its relative simplicity and has shown high inter-rater reliability for measuring adequate nurse staffing level; for example, NHPPD showed high predictive validity for patient falls [9, 19, 20]. Finally, nurses' perceptions of nurse staffing adequacy have also been used, and a prior study has revealed it to be a strong predictor of unit-acquired pressure ulcers [8].

However, despite the importance of adequate nurse staffing levels and the development of various measurement systems, no evidence supports the choice of any particular tool [13], because nurse staffing levels have constantly moving targets and the unceasing fluctuation in patient volume creates unpredictable nursing workloads [21, 22]. Therefore, in order to generate evidence to choose an appropriate nurse staffing level measurement tool, it is necessary to compare various measurement systems and examine the relationship between nurse staffing level and nurses' perceptions of nurse staffing level adequacy and fatigue on a daily basis. An effective tool can help facilitate effective nursing manager responses to relocate nursing personnel for appropriate nurse staffing level.

The current study examined the differences in the effects of nurse staffing measurement systems on nurses' perceptions of nurse staffing level, fatigue, and nursing care quality. Inadequate nurse staffing levels and increased acuity levels of patients associated with nursing workload [23] have led to an increase in fatigue [24]; moreover, fatigued nurses are more harmful to patient safety [25]. Nurse staffing level was also negatively associated with perceived nursing care quality [26].

Focused on nurse staffing levels, the current study aimed to report its influence on nurses' perceptions of inadequate nurse staffing levels and compared its effects, measured using various methods, on nurses' perceptions. Although examining nurses' perceptions of nurse staffing adequacy is one among the nurse staffing measurement methods, it is used as a dependent variable influenced by other staffing measurement variables, such as nurse-to-patient ratio and RN HPPD because it is based on nurses' perceptions.

This study aimed to determine which measurement tool best captures workload and represents nurses' perceptions. The objectives of the study were (1) to examine nurse staffing level using nurse-to-patient ratios, work intensity, and RN HPPD, according to the patient classification and number of patients in the patient groups; (2) to investigate the relationships between nurse staffing level using various measurement methods and the nurses' perceptions of nurse staffing adequacy, fatigue at the end of the shift, and quality of nursing care; and (3) to explore the factors influencing nurses' perceptions, with a focus on nurse staffing level assessed using various measurement methods.

## 2. Materials and Methods

### 2.1. Design and Participants

A cross-sectional study was conducted, and data were collected between July 18 and August 14, 2022 (four weeks). This study was conducted using the convenience sampling method in a general hospital in South Korea, and the researcher informed the nursing department about the purpose and methods of the study. The hospital comprised 14 general wards, and the study participants included registered nurses working in six wards; however, those in four comprehensive care units, one infection control ward, one hospice ward, one psychiatric ward, and one children's ward were excluded because of the heterogeneity of patient composition and nursing activities. Because the 6 selected wards were mixed-type wards with internal medicine and surgery, the patients were similar across the wards; therefore, classification by ward was not necessary. Data were analyzed based on the number of days worked to report the effects of nurse staffing level on nurses' perceptions of a work day, and data of 168 days (28 days of work across six wards) were analyzed. The sample size was determined using the *G*^*∗*^ Power 3.1.9.4 program. For multiple regression analysis, assuming the effect size = 0.15 with a significance level of 0.05 at a power of 0.80 and four predictors, the minimum required sample size was 108. Only the nursing activities of and survey completed by registered nurses were included in the analysis because the work performed by most of the nurse assistants was unit-related such as running errands, securing supplies, restocking, and environmental cleaning, and their work status was flexible, such as an assistant nurse covering more than a ward or not working at night. The study focused on registered nurses' staffing level and perceptions.

### 2.2. Data Collection

#### 2.2.1. Distributions of Nursing Personnel and Inpatients

The number of registered nurses on duty, total unique inpatients, and turnover of patients including admission, discharge, and transfer-in and transfer-out per day in six wards, which was used to measure the nurse staffing level each day, was collected for four weeks. Moreover, the daily Korean Patient Classification System-General Ward (KPCS-GW) score of every inpatient was measured by night shift nurses for the day; this was confirmed and reported to the researcher by head nurses. In Korea, the Korean Patient Classification System-1 has been used to measure the nursing care demands of inpatients in general wards, and a revised, short-form version (KPCS-GW) has been developed [27, 28]. Furthermore, the reliability and validity have been confirmed [28]. Each week, the head nurse of each ward reported the daily results to the researcher by e-mail.

#### 2.2.2. RN HPPD by Patient Classification

A work sampling method was used to identify registered nurses' nursing activities, report RN HPPD, and identify required nursing time according to patient classification. Work sampling surveys were conducted for four days (two weekdays and two weekends) during the study period for each of the six wards where nurses performed typical three-shift work: day, evening, and night. Eight trained observers, who were senior nursing students, carried out the observations in shifts and every 10 min. They recorded their observations and the activities of each registered nurse in the ward on a data collection sheet.

#### 2.2.3. Nurses' Perceptions of Nurse Staffing Level Adequacy, Fatigue, and Nursing Care Quality

At the beginning of the study, registered nurses in six wards were informed about the purpose and methods of the study, and a survey to report registered nurses' perceptions was conducted for four weeks. The questionnaires for the registered nurses, except the head nurses, were placed in each ward, and they were asked to fill out the questionnaires anonymously and drop them into enclosed questionnaire collection boxes placed in the ward after every shift for four weeks. The researcher picked up the completed questionnaires placed in the questionnaire collection boxes every week.

### 2.3. Measurements

#### 2.3.1. Patient Classification by Nursing Care Needs

Patient classifications by nursing needs were measured using the KPCS-GW, which comprises 34 items on the required nursing activities, such as vital sign checks and blood sugar test; the higher the KPCS-GW score, the higher the nursing care needs [28]. Patient groups could be classified from 1 to 4, according to the total scores of the KPCS-GW: scores of 1–10 for group 1, 11–20 for group 2, 21–30 for group 3, and 31 or more for group 4. As many patients belonged to groups 1 and 2 in the general hospital where the study was conducted, patients with scores of 1–5, 6–10, 11–15, 16–20, and over 20 were classified into groups 1, 2, 3, 4, and 5, respectively, in the analysis.

#### 2.3.2. Work Sampling

Nursing care activities were observed and reported based on the “nursing activities work sampling instrument” [29]; the authors had obtained permission to use this tool. In the present study, nursing activities were categorized as follows: 12 types in direct care work, five types in indirect care work, six types in unit-related work, and personal time. Personal time was excluded to measure the nursing hours.

#### 2.3.3. Registered Nurse Survey

The perception of adequate nurse staffing was measured using an ad hoc questionnaire comprising three items. The first item was “how adequate do you think the nurse staffing level is in the ward today to provide quality nursing care?” The responses ranged from −10 (denoting an excess, that is, too many nursing staff compared to the workload) to +10 (denoting shortage, that is, the workload was too much); 0 represented optimal workload. The higher the score, the higher the perception of insufficient nurse staffing. The second item, to measure fatigue at the end of the shift, employed a visual analogue scale for fatigue [30], ranging from 0 (not at all tired) to 10 (extremely tired). The last item—perceived nursing care quality—was measured using one item: “How do you perceive the quality of care in the ward today?” The responses were based on a 4-point Likert scale ranging from 1 (extremely poor) to 4 (excellent). Although using a single item can cause some validity and reliability concerns, global single-item indicators have been reported to provide valid and reliable measures [21, 31]. The response rate of the questionnaire was 76.8%.

### 2.4. Data Analysis

#### 2.4.1. Distribution of Nursing Personnel, Patients, and Nurses' Perceptions

Data on the average tenure of nurses in a day, number of inpatients in a ward, KPCS-GW scores of inpatients, number of patient turnover per day, and perceptions of nurses in the four weeks were analyzed through descriptive analysis. The differences between weekdays and weekends were analyzed through an independent *t*-test. *p* < 0.05 was considered statistically significant.

#### 2.4.2. Nursing Hours by Nursing Activities and RN HPPD According to Patient Classification

The number of nursing hours based on the nursing activities for four days was calculated by multiplying the number of observations for each activity performed by registered nurses by 10, because the observation was recorded every 10 min. In the case of direct nursing care, the ID number of patients receiving nursing care was recorded by the observers so that the researchers could later merge the KPCS-GW score. However, it was impossible to measure the indirect nursing care time per patient. Indirect nursing care time was allocated in proportion to the direct nursing hours because indirect care work, such as charting and handover, increases based on direct care. Accordingly, unit-related work was allocated equally for each patient [32]. RN HPPD were calculated by summing the direct care time, indirect care time, and unit-related work time, and descriptive analysis was used to report nursing hours per patient day according to the patient classification.

#### 2.4.3. Nurse Staffing Level-Related Variables

The nurse-to-patient ratio was measured by dividing the total number of inpatients by the number of nurses who had worked on a particular day. Daily work intensity per registered nurse was also reported. First, the mean value of each KPCS-GW score was calculated based on each patient group's total score, and the weighting coefficients of groups 2–5 were calculated by dividing the mean KPCS-GW scores of each patient group by the mean value of group 1. Next, the weighting coefficients were multiplied by the number of patients in each group, constituting the total sum of the nursing care intensity value. Finally, the total nursing care intensity value of the day was divided by the number of nurses who worked on that day. This method relates to the work intensity measurement system using the OPC [17, 18].

The total demanded nursing hours in a day were also calculated by the sum of the multiplied value of the number of patients in each group and the RN HPPD of the group; these hours were divided by the number of nurses who had worked on that day to measure demanded nursing hours per nurse for the day. Descriptive analysis was used to examine the nurse staffing level, and an independent *t*-test was used to examine the differences between weekdays and weekends. *p* < 0.05 was considered statistically significant.

#### 2.4.4. Associations between Nurse Staffing Level and Nurses' Perceptions

Pearson's correlation was used to examine the relationship between nurse staffing level-related variables and nurses' perceptions. Multiple regression analysis was used to examine the effects of nurse staffing level, measured using various methods, on nurses' perceptions. *p* < 0.05 was considered statistically significant. We used the variance inflation factor (VIF) for collinearity diagnostics in the study, and VIF values ranged from 1.42 to 1.47, indicating that severe collinearity did not occur between the independent variables [33]. The data were analyzed using SPSS 26.0 (IBM, SPSS Inc., Chicago, IL, USA).

### 2.5. Ethical Considerations

Before data collection commenced, the research proposal was approved by the Institutional Review Board of the corresponding author's institution (IRB no. KWNUIRB-2022-06-002-001). The researchers obtained consent from the nurses after informing them of the study purposes and methods and the reward for participation (a digital coffee coupon of approximately 70 dollars) and whether or not a nurse provided consent was not disclosed to the nursing manager. All nurses in the wards in which the study was conducted agreed to participate in the survey and received the reward after their participation.

## 3. Results

### 3.1. Distributions of Nursing Personnel, Patients, and Nurses' Perceptions

Table 1 shows the statistical distributions of nursing personnel, patients, and nurses' perceptions for four weeks. Ninety registered nurses in six wards participated in the study, and the nurses completed 1,730 shifts in four weeks. The average tenure of nurses in a day was equivalent to 3.76 (±0.85) years, and the difference between weekdays and weekends was not significant. The average number of inpatients per day in a ward was 32.95 (±8.40): 34.83 (±7.85) on a weekday and 28.27 (±7.95) on a weekend. Approximately 48.16% of inpatients had a KPCS-GW score of 10 or less, and the average KPCS-GW score was 11.56 (±7.33). The number of patient turnover per day was 11.90 (±5.83), and the difference between weekdays and weekends was significant (*p* < 0.001).

Nurses' perceived inadequacy of nurse staffing level was significantly higher (*p* < 0.001) on weekdays (3.43 ± 2.70) than on weekends (1.72 ± 2.36). Fatigue levels were also higher on weekdays (7.40 ± 1.33) than on weekends (6.12 ± 1.30). Perceived nursing care quality was higher on weekends than on weekdays, and the difference was significant (*p* < 0.001).

### 3.2. Nursing Hours by Nursing Activities and RN HPPD According to Patient Classification

Table 2 shows the nursing hours for four days based on patient classification according to nursing care needs. Nurses' working hours comprised 631.83 h (31.16%) of direct care work, 1,164.00 h (57.41%) of indirect care work, and 231.67 h (11.43%) of unit-related work. Among the tasks performed in direct care nursing hours, medication/IV administration (268.50 h, 42.50%) took the most time to execute, followed by assessment (152.33 h, 24.11%). The proportions of nursing hours for direct care during the procedures and transportation of group 5 patients (those with high nursing care needs) were 11.83 h (14.58%) and 4.00 h (4.93%), respectively, while those taken for group 1 were 4.67 h (5.03%) and 0 h (0.00%), respectively. For indirect care nursing time, data entry/retrieval through notes/computer took the most time (830.00 h, 71.31%), and it is the most common activity among all nursing activities.

The average RN HPPD was 2.35 (±1.89) h and comprised 0.73 (±0.68) h of direct care work, 1.35 (±1.21) h of indirect care work, and 0.27 (±0.08) h of unit-related work. Group 5 showed the highest RN HPPD of 3.22 (±2.65), whereas group 1 showed the lowest RN HPPD of 1.81 (±1.20). The higher the KPCS-1 score of a group, the higher the direct nursing time and RN HPPD.

### 3.3. Nurse Staffing Level Measured Using Various Methods

Table 3 shows the number of patients per registered nurse, work intensity, and demanded nursing hours for four weeks in six wards (168 days). The average nurse-to-patient ratio in a day was 1 : 3.20 (±0.76), and the difference between weekdays and weekends was not significant (*p*=0.128). The average work intensity was 11.07 (±5.33), and the difference between weekdays and weekends was also not significant (*p*=0.573).

The average demanded nursing hours for a day per nurse were 7.63 (±2.21), and the difference between weekdays and weekends was not significant (*p*=0.160). However, the proportion that demanded over 8 h was higher on the weekdays (46.67%) than it was on the weekends (29.17%), and the difference was significant (*p*=0.038).

### 3.4. Correlations of Perceived Insufficient Nurse Staffing Level, Fatigue, and Nursing Care Quality with Nurse Staffing Level-Related Variables

Table 4 shows the correlations of nurses' perceptions with nurse staffing levels using various measurement methods. The nurse staffing level-related variables, nurse-to-patient ratio, work intensity, and demanded nursing hours were correlated positively with perceived insufficient nurse staffing level and fatigue and negatively with perceived nursing care quality. Perceived insufficient nurse staffing level was correlated positively with fatigue (*r* = 0.64, *p* < 0.001) and negatively with perceived nursing care quality (*r* = −0.49, *p* < 0.001). Moreover, fatigue and perceived nursing care quality were negatively correlated (*r* = −0.55, *p* < 0.001).

### 3.5. Effects of Nurse Staffing Level on Perceived Insufficient Nurse Staffing Level, Fatigue, and Nursing Care Quality

Using multiple linear regression analysis, Table 5 shows the effects of nurse staffing level, measured using various methods, on nurses' perceptions. Whether on the weekend or not, the average tenure as a nurse among nurses who worked on a particular day and the number of turnover patients were included in the model as control variables because they were factors related to the nurse's workload or staffing level [6, 8, 34]. Moreover, nurse-to-patient ratio, work intensity per nurse, and demanded nursing hours of the day were included in different regression models to compare their effects on nurses' perceptions.

Nurses' perceptions of insufficient nurse staffing level and fatigue were lower on weekends than weekdays, and their perception of nursing care quality was higher on weekends than weekdays. Moreover, the average tenure as a nurse had a positive effect on fatigue and a negative effect on perceived nursing care quality. The nurse-to-patient ratio, work intensity, and demanded nursing hours were factors that positively affected perceived insufficient nurse staffing levels and negatively affected perceived nursing care quality. Moreover, the adjusted *R*^2^ was the highest in the model that included demand for nursing hours. Conversely, although nurses staffing level also had a positive effect on fatigue, the adjusted *R*^2^ was the highest in the model that included work intensity, in the regression model that identified the effects on fatigue (adjusted *R*^2^ = 0.31). The general linear model results for fatigue, *R*^2^ = 0.31, indicated that the variables in the model explained 31% of the variance of fatigue. The models that included the nurse-to-patient ratio showed the lowest adjusted *R*^2^ compared to those that included other nurse staffing-related variables, work intensity, and demanded nursing hours.

## 4. Discussion

The current study, conducted in a general hospital in South Korea, aimed to examine the effects of nurse staffing level measured using various methods on nurses' perceptions of nurse staffing level, fatigue, and nursing care quality. The mean score of the KPCS-GW was 11.56, and the nurse-to-patient ratio was 3.20, violating the South Korean law, which mandates 2.5 or less [35]. In a previous study conducted in South Korea, the average daily inpatients per RN in general hospitals were 2.9 [35], this nurse staffing level was slightly higher than the result of the present study.

Our study shows that more than half of the nursing activities (57.41%) were classified as indirect care work, approximately one-third (31.16%) were classified as direct care work, and 11.43% were classified as unit-related work. The results showed a lower direct care proportion (36.5%) and a higher indirect care proportion (44.9%) than a previous study conducted in the USA [36]. As nurses working in high-staffing units had significantly lower mean scores of missed care than those in low-staffing units [37], further research is needed to examine whether a low proportion of direct care leads to missed nursing care. The average of RN HPPD in the current study was 2.35, which was slightly lower than the 2.82 reported in a previous study conducted in a tertiary hospital in South Korea [32]. This may be attributed to the lower acuity of inpatients in general hospitals compared to those in tertiary hospitals. Moreover, the higher the KPCS-GW score in a group, the higher the RN HPPD, indicating that the KPCS-GW instrument reflected the actual patients' nursing care needs.

The average number of nursing care hours demanded in a day is 7.63. However, in 41.67% of the days, over eight nursing hours had been demanded, even though the fixed working hours for three-shift work were 8 h. In a previous study conducted in South Korea, the average overtime hours for nurses per shift was 1.14 h [38]. Overtime is related to a heavy workload that cannot be completed in the normal working time, and having demanded nursing hours that exceeded 8 h was related to perceived insufficient nurse staffing level, fatigue, and perceived nursing care quality in the current study.

All nurse staffing level-related variables were correlated with nurses' perceptions and affected nurses' perceptions. This means that the perception of nurses can be used to evaluate and determine the adequate nurse staffing level. In previous studies, perceived adequacy of staffing was measured using other tools to obtain accurate estimates of nurse staffing levels that reflected various factors influencing nursing workload other than patient acuity, such as cooperation, leadership, and teamwork [39, 40]. However, there has been inadequate effort to examine the actual perceived adequacy of nurse staffing for measuring optimal nurse staffing level with multiple items and to confirm psychometric properties such as reliability and criterion validity [40]. Some nurses may have considered that an optimal nurse staffing level would help them to complete tasks in a typical working time, whereas some nurses may have considered that it would help provide emotional support and education to patients. The instrument would have to present an objective criterion for an “optimal” nurse staffing level.

Even though the number of total turnover for patients and nurse staffing level-related variables were controlled, nurses' perceptions of inadequate nurse staffing level, fatigue, and perceived nursing care quality were higher on weekdays than on weekends in every regression model. In a previous study [41], nurses perceived a lower workload on weekends and holidays than on weekdays because admissions and elective surgeries are rarely scheduled on weekends/holidays except for emergency cases. Nursing managers should consider that other different factors between weekdays and weekends affect the demand of nurse staffing and allocate adequate nurse staffing.

The regression model including the nurse-to-patient ratio showed the lowest explained variability compared to other nurse staffing level-related variables. California mandated minimum nurse-to-patient ratios in hospitals because this is a very simple calculating and monitoring method; however, it is far less sensitive to the complexity of the patient mix and tends to minimize professional judgment in day-to-day staffing [13]. Therefore, measuring the nurse staffing level through a patient acuity measurement system such as the KPCS-GW or demanding nursing care time based on HPPD would be more appropriate. However, because HPPD cannot reflect the quality of care and patient factors such as age and anxiety, appropriate adjustments in the measure's application are necessary to capture variations in the characteristics of nurses, patients, and hospitals [20]. A staffing measurement system taking into account the possible factors related to nursing workload, such as the number of work interruptions and the type of working schedule, should be developed [42].

The current study has multiple strengths; it measures the actual RN HPPD at the individual patient level, compares different ways of measuring nurse staffing level, and measures nurses' perceptions on a daily basis rather than using the average of specific periods. Nevertheless, this study has certain limitations. First, the study lacks generalizability as it was conducted in a single hospital, and the preceptee's nursing work was not included in the analysis. Second, the unit-level factors that could affect the nurses' perceptions were not included in the regression model. Third, missing data related to nurses' sociodemographic information could have affected our results regarding nurses' perceptions. Finally, variables that are not considered in this study, such as the head nurse's leadership and the individual competency of each nurse, may have influenced the nurses' perceptions.

## 5. Conclusion

Nurse staffing level-related variables are factors that affect nurses' perceptions regarding the adequacy of nurse staffing level, fatigue at the end of shifts, and nursing care quality. Work intensity and RN HPPD were more reliable measurement methods than the nurse-to-patient ratio, and measuring the adequacy of nurse staffing level should reflect the acuity of inpatients and the nurses' workload. It is necessary to develop measurement instruments that ensure higher reliability and validity, considering factors influencing nurses' workload other than patient acuity, and efforts should be made to improve nurse staffing levels in clinical practice to minimize fatigue, which can lead to ill health and diminished nursing care quality.

## Figures and Tables

**Table 1:** Distributions of nursing personnel, patients, and nurses' perceptions for four weeks (*M* ± SD).

Variables	Total (overall)		Weekday (*n* = 120 days)	Weekend (*n* = 48 days)	*t* (*p*)
Nursing personnel (*n* = 90)					
Average tenure of RNs in a day (year)	3.76 ± 0.85	3.78 ± 0.84		3.72 ± 0.89	0.43 (0.667)
Distributions of patients based on unit level (*n* = 5,536)					
Number of inpatients per day in a ward	32.95 ± 8.40		34.83 ± 7.85	28.27 ± 7.95	4.87 (<0.001^*∗∗*^**)**
Group 1 (KPCS-GW score 1–5)	7.23 ± 7.02		7.24 ± 7.08	7.19 ± 6.96	0.05 (0.964)
Group 2 (KPCS-GW score 6–10)	8.64 ± 6.39		9.03 ± 6.59	7.69 ± 5.78	1.23 (0.221)
Group 3 (KPCS-GW score 11–15)	8.40 ± 4.81		9.34 ± 5.00	6.04 ± 3.29	4.22 (<0.001^*∗∗*^**)**
Group 4 (KPCS-GW score 16–20)	4.65 ± 4.72		5.16 ± 4.69	3.40 ± 4.59	2.21 (0.028^*∗*^**)**
Group 5 (KPCS-GW score **≥**21)	4.03 ± 4.98		4.06 ± 4.87	3.96 ± 5.29	0.12 (0.907)
Average scores of the KPCS-GW (weighting coefficient)	11.56 ± 7.33		11.62 ± 7.14	11.40 ± 7.89	0.97 (0.334)
Group 1 (KPCS-GW score 1–5)	3.34 ± 1.32 (1)		3.36 ± 1.32 (1)	3.29 ± 1.30 (1)	0.92 (0.359)
Group 2 (KPCS-GW score 6–10)	7.80 ± 1.40 (2.34)		7.80 ± 1.41 (2.32)	7.83 ± 1.39 (2.38)	−0.39 (0.693)
Group 3 (KPCS-GW score 11–15)	12.78 ± 1.42 (3.82)		12.77 ± 1.43 (3.80)	12.80 ± 1.40 (3.90)	−0.32 (0.749)
Group 4 (KPCS-GW score 16–20)	17.61 ± 1.34 (5.27)		17.54 ± 1.33 (5.22)	17.88 ± 1.34 (5.44)	−2.92 (0.004^*∗∗*^**)**
Group 5 (KPCS-GW score **≥**21)	24.86 ± 7.21 (7.44)		24.65 ± 7.13 (7.33)	25.39 ± 7.39 (7.73)	−1.20 (0.231)
Number of patient turnover per day	11.90 ± 5.83		14.28 ± 4.81	5.85 ± 3.27	11.03 (<0.001^*∗∗*^**)**
Admission/discharge	9.94 ± 5.59		11.98 ± 5.01	4.85 ± 3.27	9.09 (<0.001^*∗∗*^**)**
Transfer in/out	1.89 ± 1.95		2.30 ± 2.10	0.88 ± 0.89	4.53 (<0.001^*∗∗*^**)**
Perceptions of registered nurses					
Perceived insufficient nurse staffing (−10 ≤ +10)	2.95 ± 2.72		3.43 ± 2.70	1.72 ± 2.36	3.80 (<0.001^*∗∗*^**)**
Fatigue at the end of shift (0 ≤ +10)	7.04 ± 1.44		7.40 ± 1.33	6.12 ± 1.30	5.66 (<0.001^*∗∗*^**)**
Perceived nursing care quality (1 ≤ 4)	2.12 ± 0.51		2.02 ± 0.43	2.38 ± 0.60	−4.28 (<0.001^*∗∗*^**)**

*M*, mean; SD, standard deviation; *p*, level of statistical significance. ^*∗*^Correlation is significant at the 0.05 level (2-tailed). ^*∗∗*^Correlation is significant at the 0.01 level (2-tailed).

**Table 2:** Nursing hours by nursing activities and RN HPPD according to patient classification for four days (hours, %).

Variable	Overall^*∗*^	Group 1^*∗∗*^(KPCS score 1–5)	Group 2^*∗∗*^(KPCS score 6–10)	Group 3^*∗∗*^(KPCS score 11–15)	Group 4^*∗∗*^(KPCS score 16–20)	Group 5^*∗∗*^(KPCS score ≥21)
Total number of inpatients for four observed ^*∗∗∗*^days (h, %)	840	164 (19.52)	257 (30.60)	216 (25.71)	117 (13.93)	86 (10.24)
Total nursing care hours for four observed days						
Direct care (h, %)	631.83 (31.16)	92.83 (100.00)	156.83 (100.00)	188.33 (100.00)	105.00 (100.00)	81.17 (100.00)
Admission	26.67 (4.22)	2.00 (2.15)	6.17 (3.93)	12.50 (6.64)	4.17 (3.97)	1.83 (2.26)
Assessment	152.33 (24.11)	23.00 (24.78)	40.00 (25.50)	46.83 (24.87)	27.17 (25.87)	15.33 (18.89)
Hygiene	3.33 (0.53)	0.50 (0.54)	0.33 (0.21)	1.33 (0.71)	1.00 (0.95)	0.17 (0.21)
Patient mobility	1.00 (0.16)	0.33 (0.36)	0.17 (0.11)	0.33 (0.18)	0.00 (0.00)	0.17 (0.21)
Medication/IV administration	268.50 (42.50)	43.33 (46.68)	72.33 (46.12)	69.67 (36.99)	42.50 (40.48)	33.00 (40.66)
Procedures	60.67 (9.60)	4.67 (5.03)	9.00 (5.74)	22.83 (12.12)	12.33 (11.75)	11.83 (14.58)
Specimen collection/testing	20.50 (3.24)	2.33 (2.51)	5.33 (3.40)	5.67 (3.01)	3.33 (3.17)	3.83 (4.72)
Nutrition	1.50 (0.24)	0.00 (0.00)	0.17 (0.11)	0.67 (0.35)	0.17 (0.16)	0.50 (0.62)
Elimination	9.17 (1.45)	1.33 (1.44)	2.67 (1.70)	2.17 (1.15)	1.67 (1.59)	1.33 (1.64)
Transporting patient	11.67 (1.85)	0.00 (0.00)	2.17 (1.38)	3.83 (2.04)	1.67 (1.59)	4.00 (4.93)
Assisting with procedures	10.17 (1.61)	4.33 (4.67)	1.00 (0.64)	1.17 (0.62)	1.33 (1.27)	2.33 (2.87)
Patient/family interaction	66.33 (10.50)	11.00 (11.85)	17.50 (11.16)	21.33 (11.33)	9.67 (9.21)	6.83 (8.42)
Indirect care	1164.00 (57.41)					
Verbal report/handover	200.67 (17.23)					
Communication/information	103.83 (8.92)					
Room/equipment setup/cleaning	7.00 (0.60)					
Progress notes/computer: data entry/retrieval	830.00 (71.31)					
Coordination of care: rounds, team meetings	22.50 (1.93)					
Unit-related work	231.67 (11.43)					
Teaching/inservice	7.83 (0.39)					
Supplies, check, and restock	95.17 (4.69)					
Errands	46.00 (2.27)					
Meeting and administration	4.17 (0.21)					
Clerical	45.83 (2.26)					
Environmental cleaning	32.67 (1.61)					
Nursing hours per patient day (*M* ± SD)	2.35 ± 1.89	1.81 ± 1.20	2.01 ± 1.36	2.63 ± 2.10	2.83 ± 2.30	3.22 ± 2.65
Direct care	0.73 ± 0.68	0.55 ± 0.45	0.61 ± 0.49	0.84 ± 0.78	0.90 ± 0.80	1.01 ± 0.91
Indirect care	1.35 ± 1.21	1.00 ± 0.76	1.15 ± 0.87	1.51 ± 1.31	1.65 ± 1.48	1.95 ± 1.74
Unit-related work	0.27 ± 0.08	0.26 ± 0.09	0.26 ± 0.09	0.28 ± 0.08	0.28 ± 0.07	0.26 ± 0.06

Blank indicates not applicable. *M*, mean; SD, standard deviation. ^*∗*^% of column based on overall nursing activities. ^*∗∗*^% of column based on direct nursing care. ^*∗∗∗*^% of row.

**Table 3:** Nurse staffing level-related variables measured using various methods.

Category	Nurse-to-patient ratio	Work intensity			Demanded nursing hours		
	Number of patients per nurse per day (*M* ± SD)	Number of nurses worked per day (*a*, *M* ± SD)	Sum of work intensity (*b*, *M* ± SD)	Work intensity per nurse (*a*/*b*, *M* ± SD)	Demanded nursing hours per day (*c*, *M* ± SD)	Demanded nursing hours per day per nurse (*c*/*a*, *M* ± SD)	Number of days demanding over 8 nursing hours (*n*, %)
Overall (*n* = 168 days)	3.20 ± 0.76 (1.44–5.22)	10.30 ± 0.99 (8.00–13.00)	114.00 ± 55.18 (32.43–259.14)	11.07 ± 5.33 (3.60–26.88)	78.69 ± 24.01 (27.58–139.03)	7.63 ± 2.21 (3.06–13.70)	70 (41.67)
Weekday (*n* = 120 days)	3.25 ± 0.72 (1.64–5.00)	10.71 ± 0.81 (8.00–13.00)	120.35 ± 53.25 (50.69–259.14)	11.21 ± 4.83 (4.88–24.47)	83.48 ± 22.71 (38.70–139.03)	7.78 ± 2.03 (3.83–13.60)	56 (46.67)
Weekend (*n* = 48 days)	3.06 ± 0.84 (1.44–5.22)	9.25 ± 0.53 (8.00–11.00)	98.12 ± 57.24 (32.43–241.89)	10.70 ± 6.45 (3.60–26.88)	66.71 ± 23.18 (27.58–123.32)	7.25 ± 2.59 (3.06–13.70)	14 (29.17)
*t* or *χ*^2^ (*p*)	1.53 (0.128)	11.48 (<0.001^*∗∗*^)	2.39 (0.002^*∗∗*^)	0.56 (0.573)	4.30 (<0.001^*∗∗*^)	1.41 (0.160)	4.32 (0.038^*∗*^)

*M*, mean; SD, standard deviation; *χ*^2^, chi square; *p*, level of statistical significance. ^*∗*^ *p* < 0.05 (2-tailed). ^*∗∗*^*p* < 0.01 (2-tailed).

**Table 4:** Correlations of perceived nurse staffing level, fatigue, and nursing care quality with nurse staffing level-related variables (*r*,*p*).

Variables	Number of patients per nurse	Work intensity per nurse	Demanded nursing hours per nurse	Perceived insufficient nurse staffing level	Fatigue	Perceived nursing care quality
Number of patients per nurse	1					
Work intensity per nurse	0.61 (<0.001^*∗∗*^)	1				
Demanded nursing hours per nurse	0.88 (<0.001^*∗∗*^)	0.91 (<0.001^*∗∗*^)	1			
Perceived insufficient nurse staffing level	0.27 (<0.001^*∗∗*^)	0.35 (<0.001^*∗∗*^)	0.35 (<0.001^*∗∗*^)	1		
Fatigue	0.16 (0.035^*∗*^)	0.38 (<0.001^*∗∗*^)	0.32 (<0.001^*∗∗*^)	0.64 (<0.001^*∗∗*^)	1	
Perceived nursing care quality	−0.25 (0.001^*∗*^)	−0.33 (<0.001^*∗∗*^)	−0.34 (<0.001^*∗∗*^)	−0.49 (<0.001^*∗∗*^)	−0.55 (<0.001^*∗∗*^)	1

*r*, Spearman's correlation;*p*, level of statistical significance.^*∗*^Correlation is significant at the 0.05 level (2-tailed).^*∗∗*^Correlation is significant at the 0.01 level (2-tailed).

**Table 5:** Effects of nurse staffing level on perceived insufficient nurse staffing level, fatigue, and nursing care quality.

Variables	Perceived insufficient nurse staffing level			Fatigue			Perceived nursing care quality		
	Model 1 (*β*,*p*)	Model 2 (*β*, *p*)	Model 3 (*β*, *p*)	Model 1 (*β*, *p*)	Model 2 (*β*, *p*)	Model 3 (*β*, *p*)	Model 1 (*β*, *p*)	Model 2 (*β*, *p*)	Model 3 (*β*, *p*)
Weekend (vs. weekday)	−0.34 (<0.001^*∗∗*^)	−0.29 (0.002^*∗∗*^)	−0.30 (0.002^*∗∗*^)	−0.40 (<0.001^*∗∗*^)	−0.32 (<0.001^*∗∗*^)	−0.35 (<0.001^*∗∗*^)	0.31 (0.001^*∗∗*^)	0.26 (0.006^*∗∗*^)	0.26 (0.005^*∗∗*^)
Average tenure as a nurse of nurses worked in a day	0.10 (0.178)	0.08 (0.270)	0.11 (0.137)	0.16 (0.033^*∗*^)	0.17 (0.012^*∗*^)	0.18 (0.008^*∗∗*^)	−0.22 (0.003^*∗∗*^)	−0.19 (0.006^*∗∗*^)	−0.23 (0.001^*∗∗*^)
Number of turnover patients per day	−0.14 (0.136)	−0.04 (0.689)	−0.08 (0.377)	−0.04 (0.684)	0.09 (0.297)	0.02 (0.797)	0.05 (0.592)	−0.06 (0.547)	−0.01 (0.886)
Number of patients per nurse	0.26 (0.001^*∗∗*^)			0.16 (0.028^*∗*^)			−0.27 (<0.001^*∗∗*^)		
Work intensity per nurse		0.34 (<0.001^*∗∗*^)			0.41 (<0.001^*∗∗*^)			−0.35 (<0.001^*∗∗*^)	
Demanded nursing hours per nurse			0.34 (<0.001^*∗∗*^)			0.33 (<0.001^*∗∗*^)			−0.36 (<0.001^*∗∗*^)
*F* (*p* value)	7.79 (<0.001)	10.45 (<0.001)	10.47 (<0.001)	10.26 (<0.001)	19.95 (<0.001)	15.59 (<0.001)	9.83 (<0.001)	12.81 (<0.001)	13.39 (<0.001)
Adj. *R*^2^	0.14	0.19	0.19	0.18	0.31	0.26	0.18	0.22	0.23

*β*, standardized regression coefficient value; *p*, level of statistical significance; *F*, regression coefficients; *R*^2^, coefficient of determination.^*∗*^Correlation is significant at the 0.05 level (2-tailed).^*∗∗*^Correlation is significant at the 0.01 level (2-tailed).

## Data Availability

The data that support the findings of this study are available from the corresponding author upon reasonable request.
